# An overview of immune checkpoint inhibitors in breast cancer

**DOI:** 10.37349/etat.2020.00029

**Published:** 2020-12-28

**Authors:** Federica Miglietta, Maria Silvia Cona, Maria Vittoria Dieci, Valentina Guarneri, Nicla La Verde

**Affiliations:** 1 Department of Surgery, Oncology and Gastroenterology, University of Padova, 35122 Padova, Italy; 2 Division of Oncology 2, Istituto Oncologico Veneto IRCCS, 35128 Padova, Italy; 3 Department of Oncology, Luigi Sacco Hospital, ASST Fatebenefratelli Sacco, Via G.B. Grassi 74, 20157 Milano, Italy; Università Politecnica Marche, Italy

**Keywords:** Early breast cancer, metastatic breast cancer, immunotherapy, immune checkpoint inhibitors

## Abstract

Although breast cancer is not traditionally considered an immunogenic type of tumor, the combination of immunotherapy and chemotherapy has recently emerged as a novel treatment option in triple-negative subtype in the advanced setting and other similar combinations of immune checkpoint inhibitors with chemotherapy are expected to become part of the neoadjuvant management in the near future. In addition, encouraging results have been observed with the combination of immune checkpoint blockade with diverse biological agents, including anti-HER2 agents, CDK 4/6 inhibitors, PARP-inhibitors. The present review summarized the available evidence coming from clinical trials on the role of immune checkpoint inhibitors in the management of breast cancer, both in advanced and early setting.

## Introduction

Breast cancer (BC) is not traditionally considered an immunogenic tumor, especially if compared with other solid tumors as melanoma or lung cancer [1]. However, a rapidly growing body of data suggests a substantial heterogeneity in terms of immunogenicity among different BC subtypes, with hormone receptor-negative (HR-) BC being generally more immunogenic than HR-positive (HR+) tumors. Triple-negative (TN) and HER2-positive (HER2+) subtypes have been consistently reported to show higher levels of tumor infiltrating lymphocytes (TILs) as compared to the HR+/HER2-negative (HER2-) subgroup [2]. In addition, it has been shown that the presence of TILs may be capable of predicting outcome and response to treatment in early TN and HER2+ diseases, thus underlying that the biology of these aggressive BC subtypes may be deeply affected by the immune counterpart of tumor microenvironment [3–6].

Triple-negative BC (TNBC) is characterized by high genomic instability and mutational load, both of which contribute to the generation of a great amount of neoantigens, ultimately leading to increased tumor immunogenicity [7]. HER2+ disease is similarly highly immunogenic given the high proliferation activity and the overexpression of HER2, which acts as a neoantigen itself [8]. In addition, anti-HER2 monoclonal antibodies are known to mainly exert their action by activating the immune system through the mechanism of antibody-dependent cell mediated cytotoxicity (ADCC), thus enhancing the immunogenicity of this BC subtype [9, 10].

As far as HR+/HER2- BC is concerned, available evidence-although scattered-suggests that this BC subtype establishes a complex relationship with the immune system. In this context the interplay between estrogens, HR+ cancer cells, inflammatory mediators and endocrine therapies accounts, at least in part, for such complexity [11].

Based on these premises, clinical trials testing immune checkpoint inhibitors (ICIs) in BC patients, mainly focused on TN and HER2+ subtypes, have been conducted or are ongoing. Indeed, the combination of the ICI atezolizumab with chemotherapy represents the first immunotherapeutic strategy gaining the Food and Drug Administration (FDA)/European Medicines Agency (EMA) approval for the management of TN advanced BC, and other similar combinations are expected to be approved in the near future in the early setting [12]. In this context, data on potential predictive biomarkers have been generated. Notwithstanding, we currently lack a comprehensive biomarker capable of reliably selecting patients more likely to respond.

In the present review we summarized the state of art of immunotherapy for BC treatment both in early and advanced setting, focusing on activity and efficacy results of ICIs coming from clinical trials. In addition, we discussed factors possibly accounting for different results of studies conducted in similar settings, with particular emphasis on biomarker analyses.

## Early BC

### TNBC

#### Neoadjuvant setting

Fostered by the promising antitumor activity and efficacy results of clinical trials testing immunotherapy in advanced BC (as comprehensively reviewed later), several immunotherapeutic strategies have been (and are being) tested as part of the neoadjuvant management of TNBC patients (Table 1).

**Table 1.** Summary of clinical trials testing immune checkpoint inhibitors in early breast cancer (EBC), for which preliminary or final results have been presented and/or published

**Trial**	**Design**	**Setting**	**Population (*n*)**	**Drugs (*n* per arm)**	**pCR rates % (95% CI)**	**OR (95% CI), *P***	**Biomarker analysis**	**Ref.**
GeparNuevo	II Randomized	Neoadjuvant	TN (174) Any PD-L1	Durvalumab (2w)^a^ → Durvalumab + NabP → Durvalumab + EC (88)	53.4 (42.5–61.4) Window cohort: 61.0	1.45 (0.80–2.63), *P* = 0.224	sTILs and PD-L1 (trend) associated with pCR, not predictive for Durvalumab benefit	[14]
				Durvalumab (2w)^a^ → placebo + NabP → placebo + EC (86)	44.2 (33.5–55.3) Window cohort: 41.4	Window cohort: 2.22 (1.06–4.64), *P* = 0.035	iTILs (increase between baseline and end of the window phase) predictive for Durvalumab benefit (OR 9.36, 95% CI 1.26–69.65, *P* = 0.029)	
KEYNOTE-173	Ib	Neoadjuvant	TN (60) Any PD-L1	Pembrolizumab + 6 CT regimens (cohort A-F)^b^	Overall: 60 (range: 49–71)	NA	sTILs and PD-L1 associated with pCR	[16]
KEYNOTE-522	III	Neoadjuvant	TN (602) Any PD-L1	Pembrolizumab + NabP-carboplatin → A/E-C + pembrolizumab (401)	64.8 (59.9–69.5)	Estimated treatment difference: 13.6% (5.4-21.8, *P* > 0.001)	PD-L1 associated with pCR, not predictive for Pembrolizumab benefit	[17]
				Placebo + NabP-carboplatin → A/E-C + placebo (201)	51.2 (44.1–58.3)			
NeoTRIPaPDL1	III	Neoadjuvant	TN (280) Any PD-L1	Atezolizumab + NabP-carboplatin (138)	43.5 (35.1–52.2)	1.11 (0.69–1.79), *P* = 0.66^c^	PD-L1 associated with pCR, not predictive for Atezolizumab benefit	[19]
				NabP-carboplatin (142)	40.8 (32.7–49.4)			
IMpassion031	III	Neoadjuvant	TN (333) Any PD-L1	Placebo + NabP → placebo + A (168)	41.1	Delta pCR 16.5 (5.9– 27.1), *P* = 0.0044^d^	PD-L1 associated with pCR, not predictive for Atezolizumab benefit	[18]
				Atezolizumab + NabP → atezolizumab + A (165)	57.6			
I-SPY 2	II Adaptive-randomized	Neoadjuvant	HER2- (205) Any PD-L1	Pembrolizumab + paclitaxel → AC (69)	Total: 44 (33–55) ^e^ TN: 60 (44–75) ^e^ HR+: 30 (17–43) ^e^	Probability superior to control: Total: > 99.9% TN: > 99.9% HR+: 99.6%	NA	[15]
				Paclitaxel → AC (181)	Total: 17 (11–23) ^e^ TN: 22 (13–30) ^e^ HR+: 13 (7–19) ^e^			
GIADA	II	Neoadjuvant	Luminal-B (43) Any PD-L1	EC → Nivolumab + triptorelin + exemestane	16.3 (7.4–34.9) ^f^		TILs and specific immune infiltrate characteristics at baseline associated with pCR; anthracycline-induced increase in cytotoxic T-cells and decrease in T-regulatory T cells	[41]

a window phase stopped after 117 patients recruited (ethical concerns);

b CT regimens; cohort A: NabP → doxorubicin-cyclophosphamide (AC); cohort B: NabP-Carboplatin AUC6 → AC; cohort C: NabP-Carboplatin AUC5 → AC; cohort D: NabP-Carboplatin AUC2 → AC; cohort E: Paclitaxel-Carboplatin AUC5 → AC; cohort F: Paclitaxel-Carboplatin AUC2 → AC;

c pCR was a secondary endpoint;

d one-sided significance boundary *P* = 0.0184;

e estimated rates of pCR;

f 2-step statistical design: second step (8 pCR/43 patients) not met;

w: weeks; NA: not available; pCR: pathologic complete response; OR: odds ratio; EC: epirubicin-cyclophosphamide; sTILs: stromal TILs; iTILs: intra-tumoral TILs; AC: doxorubicin-cyclophosphamide

The GeparNuevo double-blind phase II trial randomized 174 cT2-cT4 TNBC patients to receive either the anti-programmed death-ligand 1 (PD-L1) agent Durvalumab or placebo in combination with nab-paclitaxel (NabP), followed by dose-dense EC [13]. In the window-(of-opportunity)-phase, either durvalumab or placebo were administered for 2 weeks as single agents with the aim of priming the immune system before the start of chemotherapy (it should be noted that due to ethical concerns regarding a possible delay in the start of chemotherapy, after 117 patients enrolled, window-phase was prematurely stopped following a trial amendment). pCR in both breast and lymph-nodes was the primary endpoint. The combination of durvalumab plus chemotherapy was reported to numerically (but not significantly) increase pCR rates as compared to the placebo arm (53.4% *vs.* 44.2%, OR 1.45, 95% CI 0.80–2.63, *P* = 0.284). In addition, among patients treated in the window-phase cohort, those receiving durvalumab experienced a significantly increase in pCR rates as compared to those treated with placebo (61.0% *vs.* 41.4%, OR 2.22, 95% CI 1.06–4.64, *P* = 0.035). These data overall suggest that induction with durvalumab before the combination therapy may enhance the immunological anti-tumor activity. However, differences in composition of patients between window-phase and not-window phase cohorts (window phase cohort was enriched for patients with lymph-node involvement and stage ≥ IIa disease) may be taken into account in the interpretation of these results. Biomarker analyses revealed that higher sTILs were significantly and positively associated with pCR in the overall cohort but were not specifically predictive for durvalumab benefit. Interestingly, the magnitude of benefit with durvalumab was significantly and independently larger in patients showing an increase in iTILs between baseline and the end of the window phase (multivariate analysis: OR 9.36, 95% CI 1.26–69.65, *P* = 0.029; interaction test, *P* = 0.085). Although a trend in increased pCR rates was also observed in PD-L1 positive patients, the amount of pCR benefit from durvalumab was not affected by PD-L1 status (assessed on both tumor and immune cells using Ventana SP263 antibody). Similarly, TIL- and IFN-gamma mRNA signatures were significantly associated with pCR, without specifically predicting durvalumab benefit. On the other hand, the upregulation of 14 immune-related genes was reported to be predictive for increased pCR rates specifically in the durvalumab arm [14].

The I-SPY 2 trial is an adaptive randomized phase II trial for patients with stage II/III BC comparing diverse targeted agents in combination with chemotherapy *versus* chemotherapy alone as neoadjuvant treatment in BC patients, adopting pCR on both breast and lymphnodes as primary endpoint [15]. Based on a predictive probability of success in a confirmatory phase III trial (graduation for efficacy: ≥ 85% probability of success in a randomized phase III trial including 300 patients), the anti-PD1 ICI pembrolizumab in combination with paclitaxel, followed by AC, graduated in BC patients with TN biomarker signature. In this patients’ subgroup, the addition of pembrolizumab determined a three-fold increase in the estimated pCR rates as compared to chemotherapy alone (pCR rates 60% *vs.* 22% with a probability of pembrolizumab plus chemotherapy being superior to chemotherapy alone = 99.6% and a predictive probability of success in a phase III trial = 83.4%). No new safety concerns in relation to pembrolizumab emerged from the I-SPY 2 trial.

In the phase Ib open label multi-cohort KEYNOTE-173 trial the association of pembrolizumab and different chemotherapy regimens (taxane-either paclitaxel or NabP+/– carboplatin, followed by EC) was tested in 60 high-risk TN early BC patients (T1c, N1-N2; T2-T4c, N0-N2) [16]. pCR was the primary endpoint, while objective response rate (ORR), event-free survival (EFS) and overall-survival (OS) represented secondary endpoints. The overall pCR rate (ypT0/is N0) was 60%, ranging from 30% to 80% across cohorts. No unexpected toxicities were observed. Exploratory analyses revealed that higher pre- and on-treatment sTILs and higher pre-treatment PD-L1 expression [based on the combined positive score (CPS), defined as the number of PD-L1 stained cells including tumor cells, lymphocytes, and macrophages/histocytes, divided by the total number of viable tumor cells and multiplied by 100, and assessed using the IHC 22C3 pharmDx assay] were significantly and positively associated with pCR. The lack of a control arm did not allow to draw conclusions on the possible predictive role of these biomarkers in terms of pembrolizumab plus chemotherapy activity.

The more recent phase III KEYNOTE-552 trial randomized 602 stage II or III TNBC patients to receive the combination of chemotherapy (carboplatin-paclitaxel followed by EC) with either pembrolizumab or placebo [17]. pCR rates were significantly higher in the chemotherapy plus pembrolizumab arm as compared to the chemotherapy plus placebo arm (64.8% *vs.* 51.2%, estimated treatment difference 13.6%, 95% CI 5.4–21.8, *P* < 0.001). Follow up period was not sufficiently long to show survival differences, however in the first EFS interim analysis, 18-month EFS was numerically longer in the pembrolizumab containing arm as compared to placebo containing arm (91.3% *vs.* 85.3%, HR 0.63, 95% CI 0.43–0.93). PD-L1 positive status (defined as CPS > 1 as assessed by the IHC 22C3 pharmDx assay) was associated with increased rates of pCR both in patients treated with pembrolizumab and in patients treated with placebo, and did not resulted predictive for the efficacy of pembrolizumab (pCR rates in PD-L1 positive subgroup: 68.9% *vs.* 54.9% in pembrolizumab plus chemotherapy *vs.* placebo plus chemotherapy, respectively, delta-pCR = 14.2; 5.3–23.1; pCR rates in PD-L1 negative subgroup: 45.3% *vs.* 30.3% in pembrolizumab plus chemotherapy *vs.* placebo plus chemotherapy, respectively, delta-pCR = 18.3; –3.3–36.8). On the other hand, the magnitude of pCR benefit derived from the addition of pembrolizumab to neoadjuvant chemotherapy was larger in node positive BC patients (delta-pCR between pembrolizumab and placebo by lymph-node involvement: 6.3% *vs.* 20.6% in node negative and node-positive, respectively). This finding generated the hypothesis that in the presence of nodal disease, the administration of ICIs may boost the immune-priming phase in lymph nodes, generating the hypothesis that the presence of nodal disease could identify patients who are more likely to benefit from the addition of immunotherapy to chemotherapy.

In the recently presented IMpassion031 phase III trial, 333 patients with TN primary BC > 2 cm were randomized to receive the combination of either atezolizumab + NabP followed by atezolizumab + Doxorubicin or Placebo + NabP followed by Placebo + Doxorubicin in the neoadjuvant setting [18]. The primary endpoint was pCR in the intention-to treat population (ITT) population and PD-L1 positive subgroup, defined as PD-L1 expression on immune cells ≥ 1% by Ventana SP142 assay (adaptive enrichment design). The study showed a significant increase in pCR rates in the immunotherapy-containing arm as compared to the control arm in the ITT population (pCR rates in atezolizumab + chemotherapy arm *vs.* Placebo + chemotherapy arm: 57.6% *vs.* 41.1%, delta pCR 16.5%, *P* = 0.0044 with one-sided significance boundary *P* = 0.0184), regardless of PD-L1 status (delta pCR between atezolizumab + chemotherapy *vs.* Placebo + chemotherapy in PD-L1 positive subgroup: 19.5%, *P* = 0.021 which did not cross significance boundary of 0.0184). Notably, subgroup analysis revealed a larger magnitude of benefit in terms of pCR in node-positive subgroup as compared to node-negative subpopulation (delta pCR in node-positive and node-negative subgroups: 26.6%, 95% CI 9.8–43.4, and 8.8%, 95% CI –4.8–22.5).

The NeoTRIPaPDL1 study is an open-label phase III trial randomizing 280 TNBC patients (cT1cN1, cT2N1, cT3N0) to receive chemotherapy with carboplatin plus NabP with or without the anti-PD-L1 agent atezolizumab. Anthracycline-based chemotherapy was administered postoperatively [19].

EFS was the primary endpoint (immature data), while pCR was one of the secondary endpoints. Although this study was not powered to show pCR rate differences, the addition of atezolizumab to chemotherapy failed to significantly increase pCR rates (43.5% *vs.* 40.8% in atezolizumab *vs.* no-atezolizumab arm, OR 1.11, 95% CI 0.69–1.79, *P* = 0.66). PD-L1 positive expression (as defined by at least 1% of total tumor area occupied by positive immune cells by using the Ventana SP142 IHC assay) positively influenced pCR rates in the overall cohort and in each treatment arm but was not specifically predictive for atezolizumab benefit in terms of pCR.

Differences in the composition of KEYNOTE-522, IMpassion031 and NeoTRIP trial populations may have contributed to conflicting findings in terms of immunotherapy efficacy. Indeed, the NeoTRIP trial population was enriched for patients with higher disease burden (node-positive and/or stage III BC patients) as compared to the KEYNOTE-522 and IMpassion031 trials, which were conversely enriched for node-negative, stage II BC patients. However, as already mentioned, subgroup analyses of both KEYNOTE-522 and IMpassion031 trials showed a larger magnitude of benefit from immunotherapy in node-positive, thus downsizing the contribution of different patient features in affecting these conflicting results.

Secondly, chemotherapy backbones were different. Indeed, results from clinical trials testing immunotherapy in ABC were overall more promising in terms of larger activity and increased efficacy, when ICIs were combined with chemotherapy (as reviewed later), coherently with the well-established notion that chemotherapy exerts its action, at least in part, by modulating the immune system [20]. Interestingly, it has been suggested that anthracyclines may enhance tumor microenvironment sensitivity to PD1/PD-L1 blockade. In particular, in the adaptive phase II TONIC trial, which tested several induction strategies for immunotherapy in 67 TN metastatic breast cancer (MBC) patients, doxorubicin followed by the anti-PD1 Nivolumab was associated with the highest ORR (35%) [21]. Short-term induction with doxorubicin was reported to be capable of upregulating immune-related genes involved in T-cell cytotoxicity and PD1/PD-L1 pathways. It may therefore be speculated that the choice of the chemotherapy backbone for immunotherapy, especially in terms of anthracycline omission (KEYNOTE-522 and IMpassion031 designs included anthracyclines as part of the neoadjuvant treatment, while the NeoTRIP trial did not), may have played a crucial role in this context.

Finally, the choice of the ICI may have contributed to the negative results of the NeoTRIP trial. Indeed, atezolizumab targets PD-L1, thus potentially allowing PD-L2 to exert a residual immunosuppressive activity, as opposed to pembrolizumab which inhibits PD1. However, the choice of the ICI may not be sufficient to explain the negative results from the NeoTRIP trial, given the fact that the positive IMpassion031 trial likewise adopted atezolizumab as ICI to be associated to standard neoadjuvant chemotherapy.

Another point that deserves further discussion is the different role of PD-L1 status in predicting the benefit from ICIs that derives from studies in the neoadjuvant and in the metastatic setting. Overall, clinical trials testing the combination of ICIs with neoadjuvant chemotherapy in TNBC patients consistently failed to show a predictive value of PD-L1 in determining immunotherapy benefit. These findings appear in contrast with the IMpassion130 trial results, where the benefit deriving from the addition of atezolizumab to chemotherapy as first-line treatment of TN MBC patients was mostly limited to the PD-L1 positive subgroup. A possible explanation for such inconsistency is that PD-L1 may not be the most appropriate predictive biomarker for immunotherapy benefit, mostly due to the significant heterogeneity in terms of type of assays, cutoffs for PD-L1 positivity, scoring systems adopted to define PD-L1 status across clinical trials, and dynamic changes [22]. Interestingly, a recent analysis of more than 7, 000 cancer patients from the The Cancer Genome Atlas (TCGA) revealed that PD-L1 was only weakly associated with the magnitude of benefit from PD1/PD-L1 blockade, while the abundance of CD8+ T cells, tumor mutational burden (TMB) and the fraction of samples with high PD1 mRNA expression were more strongly predictive for the response to anti-PD1/PD-L1 agents [23]. Moreover, the biologic meaning of PD-L1 expression may be different in the early *vs.* the metastatic setting. Early-phase clinical trials in the metastatic setting have pointed out that the efficacy of immunotherapy is higher in first line as compared to later lines. The level of immune infiltrate tends also to decrease from primary tumor *vs.* matched metastases. Based on these considerations, it is rationale to postulate that the immune microenvironment of early disease may be more susceptible to immune-activation, whereas the microenvironment of advanced disease might be more inert to immune-stimulating therapies [24–28].

#### Adjuvant/post-neoadjuvant setting

Although no data are currently available on the possible role of ICIs in the adjuvant setting, results from clinical trials currently ongoing are awaited (NCT02926196; NCT02954874; NCT03498716). However, it should be mentioned that adjuvant trials typically require large populations, long-term follow up periods, and, as a result, extensive financial resources, to capture small differences in disease-free survival rates. In this context, the post-neoadjuvant setting may represent a more strategical platform to test the efficacy of novel or escalated treatment strategies, like ICIs for high-risk TNBC patients. Indeed, the positive results from CREATE X [29] and KATHERINE [30], that evaluated capecitabine and T-DM1, respectively, as adjuvant strategies in patients failing to achieve pCR, highlighted that the inclusion of a more selected group of high-risk patients based on the presence of residual disease after neoadjuvant treatment may allow for a more rational clinical positioning of new anti-cancer agents. This kind of approach has also been endorsed by FDA [31]. Indeed, whilst the achievement of pCR after NACT represents a well-established surrogate for long-term outcome, the presence of residual disease after neoadjuvant chemotherapy might allow the identification of TNBC patients at higher risk of relapse, for whom chemotherapy alone could represent a suboptimal treatment [32]. This notion strengthens the rationale for evaluating whether immunotherapy given as post-neoadjuvant treatment may enhance prognosis of high-risk TNBC patients. Two ongoing trials are currently ongoing in this setting. In detail, the A-BRAVE trial (NCT02926196) is currently enrolling in two strata patients with TNBC considered at high risk after completing the standard treatment with curative intent chemotherapy and surgery. Patients in stratum A are enrolled after primary surgery followed by adjuvant chemotherapy; stratum B includes patients who received neoadjuvant chemotherapy and surgery without achieving a pCR. The randomization is 1:1 between the anti-PD-L1 agent Avelumab for 1 year or observation. Similarly, the SWOG 1418 trial (NCT02954874) is currently randomizing TNBC patients who failed to achieve pCR after neoadjuvant chemotherapy to receive either pembrolizumab or observation in the post-neoadjuvant setting.

### HER2+ EBC

Both preclinical and clinical evidence supports the synergistic effect of ICIs and anti-HER2 agents. Indeed, it has been reported that the administration of anti-PD1 monoclonal antibody (mAb) in immunocompetent mice could enhance the therapeutic activity of anti-HER2 mAb by enhancing the adaptive anti-tumor immune response [33].

Similarly, the combination of T-DM1 and anti PD1/CTLA4 agents fostered both innate and adaptive immune response, ultimately resulting in tumor rejection in a HER2-expressing orthotopic tumor model [34].

Interestingly, as reviewed later, early-phase clinical trials testing diverse combinations of ICIs plus anti-HER2 agents in advanced HER2+ BC led to promising results [35–37]. Based on these premises, clinical trials testing immunotherapy in the early setting, mostly as neoadjuvant strategy in BC patients with HER2+ disease have been initiated (NCT03595592; NCT03726879; NCT03515798) and results are pending.

### HR+/HER2- EBC

As already mentioned, HR+/HER2- BC is traditionally considered less immunogenic as compared to HER2+ and TNBC. However, available evidence suggests that HR+ BC cells may rely on subtler mechanisms to interact with the immune microenvironment, as compared to more aggressive BC subtypes [11]. In addition, among HR+/HER2- tumors, those with luminal B-like phenotype should be considered, also from an immunological point of view, a distinct entity from luminal A-like BC. Indeed, luminal B-like BC is characterized by high mutational load, thus possibly enhancing the anti-tumor immune response. In addition, although available evidence on the clinical relevance of the immune infiltrate in this HR+/HER2- is overall inconclusive, results from a recent large case-cohort study suggest that the prognostic value of this biomarker may be restricted to the subgroup of patients exhibiting a Luminal B phenotype [38].

Interestingly, in the abovementioned adaptive phase II I-SPY 2 trial, the combination of pembrolizumab plus standard neoadjuvant chemotherapy graduated in all HER2- signatures, including HR+/HER2-, by more than doubling the estimated pCR rates as compared to chemotherapy alone in this subpopulation (estimated pCR rates: 34% *vs.* 13%).

Finally, endocrine therapy may represent an additional player in this complex interplay between HR+/HER2- disease and immune system. Indeed, it has been reported that aromatase inhibitors may promote a cytotoxic polarization of tumor immune microenvironment by increasing CD8/FOXP3 T cell ratio, thus providing an appealing rationale to test combination strategies consisting of cytotoxic agents, endocrine therapy and ICIs [39, 40].

Interestingly, in the multicenter phase II GIADA trial (Eudract 2016-004665-10), 43 premenopausal HR+/HER2-luminal B-like BC patients were enrolled to receive, in the neoadjuvant setting, induction anthracycline-based chemotherapy followed by the combination of endocrine therapy (ovarian-function suppression + aromatase inhibitor) plus the anti-PD1 agent Nivolumab [41].

Although the trial did not meet its primary endpoint (2nd stage: ≥ 8 pCR/43), translational analyses revealed increased pCR rates in patients with high sTILs and specific immune infiltrate characteristics at baseline; in addition, anthracycline-based induction chemotherapy was suggested to be potentially capable of reverting the immunosuppressive tumor microenvironment of luminal BC by increasing cytotoxic T cells, while decreasing the T-regulatory counterpart. Overall, available evidence provides interesting insights for a rational and strategical designing of future immunotherapy trials in this BC subtype.

Indeed, results from the ongoing Checkmate 7FL phase III randomized trial, investigating the combination of neoadjuvant chemotherapy (taxane with or without anthracyclines) and post neoadjuvant endocrine therapy plus either placebo or Nivolumab in patients with high-risk HR+/HER2- BC are awaited.

## Metastatic BC (MBC)

MBC remains to date an incurable disease, whose prognosis is closely related to different factors, such as biological subtype, organ involvement, treatments options. The role of ICIs in metastatic BC is still under study, with many hopes and few sureness (Table 2).

**Table 2.** Phase II and III published study with immune checkpoint inhibitors in MBC, for which preliminary or final results have been presented and/or published

**Trial**	**Design**	**Line of therapy**	**Population (*n*)**	Drugs/ich test*	**ORR% (95% CI)**	**Median PFS months (95% CI)**	**Median OS months (95% CI)**	**Ref.**
KEYNOTE-086 (cohort B)	II	1	TNBC PD-L1+ (84)	Pembrolizumab	18 (13.9–31.4)	2.1 (2.0–2.2)	18 (12.9–23.0)	[44]
				DAKO 22C3 pharmDx				
KEYNOTE-086 (cohort A)	II	> 2	TNBC any PD-L1 (170)	Pembrolizumab	Total 5.3 (2.7–9.9)	Total 2 (1.9–2.0)	Total 9 (7.6–11.2)	[43]
				DAKO 22C3 pharmDx	PD-L1+ 5.7 (2.4–12.2)	PD-L1+ 2 (1.9–2.1)	PD-L1+ 8.8 (7.1–11.2)	
KEYNOTE-119	III	> 1	TNBC any PD-L1 (622)	Pembrolizumab (P) *vs.* CT^§^	Total P: 2.1 (6.6–13.4)	Total P: 2.1 (2.0–2.1)	Total P: 9.9 (8.3–11.4)	[48]
				DAKO 22C3 pharmDx	CT: 3.3 (7.4–14.6)	CT: 3.3 (2.7–4.0)	CT: 10.8 (9.1–12.6)	
					CPS ≥ 10 P: 17.7 (10.7–26.8) CT: 9.2 (4.3–16.7)	CPS ≥ 10 P: 2.1 (2.0–2.5) CT: 3.4 (2.3–4.1)	CPS ≥ 10 P: 12.7 (9.9–16.3) CT: 11.6 (8.3–13.7)	
					CPS ≥ 1 P: 12.3 (8.1–17.6) CT: 9.4 (5.8–14.3)	CPS ≥ 1 P: 2.1 (2.0–2.1) CT: 3.1 (2.3–4.0)	CPS ≥ 1 P: 10.7 (9.3–12.5) CT: 10.2 (7.9–12.6)	
IMpassion130	III	1	TNBC any PD-L1 (902)	Atezolizumab + NabP *vs.* NabP + placebo	Experimental arm PD-L1+ 58.9 (51.5–66.1)	Experimental arm PD-L1+ 7.5 (6.7–9.2)	Experimental arm PD-L1+ 25 (19.5–30.7)	[49]
				VENTANA SP142	Total 56.0 (51.3–60.6)	PD-L1-5.6 (5.5–7.3)	PD-L1-19.6 (16.3–21.6)	
					Control arm PD-L1+ 42.6 (35.4–50.1) Total 45.9 (41.2–50.6)	Control arm PD-L1+ 5.3 (3.8–5.6) PD-L1-5.6 (5.4–7.3)	Control arm PD-L1+ 18 (13.6–20.1) PD-L1-19.6 (16.9–22.2)	
IMpassion131	III	1	TNBC any PD-L1 (651)	Atezolizumab + paclitaxel *vs.* paclitaxel + placebo	Experimental arm PD-L1+ 55 (45–65)	Experimental arm PD-L1+ 5.7 (5.4–7.2)	Experimental arm PD-L1+ 28.3 (19.1–NA)	[50]
				VENTANA SP142	ITT 47 (41–54)	ITT 5.6 (5.4–6.5)	ITT 22.8 (17.1–28.3)	
					Control arm ITT 63 (56–70)	Control arm ITT 6.0 (5.6–7.4)	Control arm ITT 22.1 (19.2–30.5)	
KEYNOTE-355	III	1	TNBC any PD-L1 (847)	Pembrolizumab (P) + CT^§^ *vs.* CT^§^ + placebo	NA	ITT 7.5 (P + CT) 5.6 (CT)	NA	[51]
				DAKO 22C3 pharmDx		CPS ≥ 10 9.7 (P + CT) 5.6 (CT)		
						CPS ≥ 1 7.6 (P + CT) 5.6 (CT)		
NCT03051659	II	≥ 2 lines of hormonal therapies and 0–2 lines of CT	HR+/HER2-	Eribulin mesylate (E) with or without pembrolizumab (P)	P + E: 25 E: 34	PD-L1+ P + E 4.1 (1.8–8.5) E 4.2 (2.8–5.9)	NA	[54]
				DAKO 22C3 pharmDx		TIL > 10% P + E 3.2 (0.4–NA) E 4.1 (2.3–NA)		
						NLR > 4 P + E 5.2 (2.0–6.4) E 4.1 (2.1–4.6)		
						TMB > 6 P + E 4.1 (2.1–5.8) 0 E 3.9 (1.6–6.2)		
PANACEA	Ib/II	> 1 trastuzumab-based therapy	HER2+ trastuzumab-resistant (6 + 52) Any PD-L1	Pembrolizumab + trastuzumab	Phase II	Phase II	Phase II	[35]
				DAKO 22C3 pharmDx	PD-L1+ 15 (7–29) PD-L1– 0 (0–18)	PD-L1+ 2.7 (2.6–4.0) PD-L1-2.5 (1.4–2.7)	PD-L1+ Not Reached PD-L1-7 (4.9–9.8)	
KATE2	II	> 1 taxane and trastuzumab-based therapy or within 6 mounths of adjuvant therapy	HER2+ (133) Any PD-L1	Atezolizumab (A) + trastuzumab emtansine (T-DM1) *vs.* placebo (p) + T-DM1	Total A + T-DM1: 45 T-DM1 + p: 43	Total A + T-DM1: 8.2 T-DM1 + p: 6.8	NA	[36, 37]
				VENTANA SP142	PD-L1+ A + T-DM1: 54 T-DM1 + p: 33	PD-L1+ A + T-DM1: 8.5 T-DM1 + p: 4.1		
					PD-L1- A + T-DM1: 40 T-DM1 + p: 50	PD-L1- A + T-DM1: 6.8 T-DM1 + p: 8.2		
TOPACIO	II	0–2	TNBC (55)	Niraparib + pembrolizumab	21 (12–33)	8.3 (2.1-NA)	NA	[63]
				DAKO 22C3 pharmDx	BRCA mutated 47 (24–70)			
MEDIOLA	II	> 1	BRCA mutetd, HER2- (30)	Olaparib + durvalumab	Total 63.3 (43.9–80.1)	Total 8.2 (4.6–11.8)	Total 20.5 (16.2–23.9)	[62]
			TNBC (17)	VENTANA SP263	No prior lines 78 (40.0–97.2)	No prior lines 9.9 (2.2–13.8)	No prior lines 21.3 (9.0–NA)	
					1 prior line 64 (30.8–89.1)	1 prior line 11.7 (1.9–NA)	1 prior line 22.7 (10.1–NA)	
					2 prior lines 50 (18.7–81.3)	2 prior lines 6.5 (1.0–8.2)	2 prior lines 16.9 (4.6–NA)	

* ICH ASSAY: immunoistochemistry assay emplyed for PDL-1 status evaluation;

§ Chemotherapy according physician choice; NA: not available; CT: chemotherapy; CTX: cyclophosphamide; CDDP: cisplatin; ORR: overall response rate; HR: hormon receptor; CPS: PD-L1 combined positive score; TIL: tumor-infiltrating lymphocytes; NLR: neutrophil-lymphocyte ratio; PFS: progression free survival

Among different BC subtypes, TNBC has the worst prognosis and limited treatment options, mainly based on chemotherapy. On the other hand, the greater sTIL and iTIL levels and the higher TMB and PD-L1 expression, make it a particularly suitable candidate for immunotherapy.

The results of initial trials testing ICIs as single agent for TN MBC showed modest ORR that were higher in the first line setting (up to 24%) as compared to patients who received immunotherapy in later lines [42–45].

A second category of early studies evaluated combinations of chemotherapy and ICIs in advanced TNBC. The combination of atezolizumab + NabP led to 39% of overall responses, that were higher in patients treated in the first line (54%) and in PD-L1 positive patients (42%) [46].

The combination of pembrolizumab and eribulin-mesylate induced a 26% rate of overall response, which again was numerically higher in the first-line cohort [29% *vs.* the second/third-line cohort (22%)] [47]. Overall, the two key messages emerging from these early studies were: patients in the first-line setting may be more sensitive to immunotherapy-based treatment, and combination with chemotherapy may enhance the efficacy of immunotherapy. As a further confirmation of these early findings, the KEYNOTE-119 trial, that randomized TNBC patients previously treated with 1-2 prior treatments for metastatic disease to receive pembrolizumab *vs.* chemotherapy of physician’s choice, did not demonstrate any significant difference in OS between the two arms in patients with CPS > 10 or > 1 or in all patients, although the effect of pembrolizumab treatment was larger as the level of PD-L1 expression increased [48].

The TONIC trial evaluated different induction strategies to enhance the efficacy of the anti-PD1 Nivolumab given as single-agent in TN advanced BC patients. This trial enrolled patients who received no more than 3 lines for advanced disease. Patients were randomized to receive different 2-weeks induction treatments (irradiation, CTX, CDDP, doxorubicin or control) followed by nivolumab. The results showed that the cohort of patients who received induction with doxorubicin achieved the highest overall response rate (35%), confirming that chemotherapy may be able to convert a cold microenvironment into a hot one, more susceptible to the mechanism of action of immunotherapy and that anthracyclines, by inducing immunogenic cell death, are optimal candidate drugs in this context [21].

Atezolizumab was the first ICI to be approved for the treatment of MBC based on the IMpassion130 trial results. This trial enrolled about 900 patients with metastatic TNBC disease who received no prior therapy for advanced disease (prior chemotherapy for early BC was allowed if relapse occurred at least 12 months after treatment conclusion). Patients were randomized to receive NabP plus atezolizumab or NabP plus placebo. The association of the PD-L1 antibody atezolizumab with NabP significantly improved PFS in the intention-to-treat population: median PFS 7.2 months *vs.* 5.5 months (HR 0.80, 95% CI 0.69–0.92, *P* = 0.002). Among patients with PD-L1 positive tumors (at least 1% of total tumor area occupied by positive immune cells by using the Ventana SP142 IHC assay), the median PFS was 7.5 months and 5.0 months, respectively (HR 0.62, 95% CI 0.49–0.78, *P* < 0.001) [12]. At the last interim analysis, no significant difference in OS was observed in the intention-to-treat population between the two arms, whereas a 7-months improvement in median OS was observed in PD-L1 positive patients treated with atezolizumab plus NabP (25.0 months) *vs.* placebo plus NabP (18.0 months, HR 0.71, 95% CI 0.54–0.93, statistical significance was not formally tested according to the hierarchical statistical plan) [49]. Toxicities were acceptable and consistent with the expected safety profiles of each agent and previous studies. In the experimental arm, 29 (6.4%) patients had adverse events that led to the discontinuation while fatal adverse events occurred in 6 (1.3%) cases. IMpassion130 has been a practice changing study establishing the new standard of care for previously untreated PD-L1 positive TN MBC patients. This first-line combination is approved by FDA and EMA in TNBC patients who present a PD-L1 expression > 1% on immune cells.

At the recent European Society for Medical Oncology (ESMO) Virtual Congress 2020, results from the phase III IMpassion131 were presented. This is the IMpassion130 “twin study” in which authors aimed to evaluate, in a similar population, the role of backbone chemotherapy in association with atezolizumab. The trial did not meet its primary endpoint, where atezolizumab plus paclitaxel as first line treatment failed to improve outcomes in PD-L1 positive MBC patients. In detail, PFS was not significantly improved by atezolizumab plus paclitaxel *vs.* paclitaxel alone in both PD-L1 positive (6.0 months *vs.* 5.7 months, HR = 0.82, *P* = 0.20) and ITT population (5.7 months *vs.* 5.6 months, HR = 0.86). The combination also did not improve OS in PD-L1 positive group, with even a trend towards decreased OS (22.1 months *vs.* 28.3 months, HR = 1.12; ITT: 19.2 months *vs.* 22.8 months, HR = 1.11), but moderately affected RR (63.4% *vs.* 55.4% among PD-L1 positive patients and 53.6% and 47.5% in the overall population). Although the reasons for these divergent results from IMpassion130 and IMpassion131 are largely unknown, a possible explanation may rely on the larger use of steroid premedication in IMpassion131 as compared to IMpassion130 trial. Notably, when looking at trial population composition and features, no other relevant differences in terms of sample size, DFIs, number of metastatic sites, prior chemotherapy for EBC and/or ABC, or proportion of PD-L1 positive patients were observed. Finally, PD-L1 may be considered a suboptimal predictive factor in this setting, thus not probably representing the ideal biomarker to select patients for ICIs. Overall considered, these data underline the need to better understand the interactions cancer-immune system, the more suitable combination between ICI and backbone chemotherapy [50].

Interesting finding emerged from the KEYNOTE-355, a phase III trial investigating the combination of another ICI, pembrolizumab, plus chemotherapy (NabP; paclitaxel; or gemcitabine/carboplatin) *vs.* placebo plus chemotherapy in TN MBC patients. According to the inclusion criteria, patients should have not received any prior treatment for metastatic disease and relapse should have occurred at least 6 months after the conclusion of chemotherapy for early BC. Eight hundred forty-seven patients were randomized 2:1; the primary endpoint was PFS in PD-L1 positive patients (CPS > 10 and > 1, assessed with 22C3 pharmDx assay) and in the intention-to-treat population, to be tested according to a hierarchical statistical design. Notable, PD-L1 status was assessed using a different ICH test compared to IMpassion130-131, where Ventana SP142 was employed, and evaluated not only in the immune compartment but also in tumor cells (CPS). With regards to the first PFS endpoint, the statistical assumption was met and pembrolizumab plus chemotherapy significantly improved PFS *vs.* chemotherapy plus placebo in pts with CPS ≥ 10 tumors (median 9.7 months *vs.* 5.6 months, HR 0.65, 95% CI 0.49–0.86, *P* = 0.0012). No statistically significant benefit of pembrolizumab + chemotherapy was observed in patients with CPS ≥ 1 tumor (median PFS 7.6 months *vs.* 5.6 months, HR 0.74, 95% CI 0.61–0.90, *P* = 0.0014, with a pre-specified *P* value boundary of 0.00111). According to the statistical plan, PFS analysis in the intention-to-treat population was only descriptive and reported a median PFS of 7.5 with pembrolizumab *vs.* 5.6 with placebo [51].

Although the best results with ICIs have been achieved in TNBC, significant evidence of immunogenicity have been proven also in HER2+ MBC. As already mentioned, the hypothesis is that ICIs and anti-HER2 agents could be synergic and that their combination could overcome potential resistance. The phase Ib/II PANACEA trial tested the efficacy and safety of pembrolizumab in combination with trastuzumab, in advanced HER2+ trastuzumab-resistant MBC. In PD-L1 positive tumors the combination was active and safe with a durable clinical benefit. In this cohort an ORR of 15% and a disease control rate (DCR) of 25% were detected. Activity was higher in the ≥ 5% TILs subgroup with ORR and DCR of 39% and 47%, respectively. No objective responses were observed in the PD-L1 negative cohort. PANACEA study provides proof of principle evidence that immunotherapy can reverse trastuzumab resistance in metastatic HER2+ BC [35].

The recent KATE2 study evaluated the addition of atezolizumab to T-DM1 in 202 patients with locally advanced or metastatic HER2+ BC patients priorly treated with trastuzumab and taxane-based therapy. Overall median PFS was 8.2 months in the atezolizumab group *vs.* 6.8 months in the placebo group with no statistically significant difference (HR 0.82, 95% CI 0.55–1.23, *P* = 0.33). In the intention-to-treat population, 1-year OS was similar in both arms. In the PD-L1 positive subgroup, both PFS and OS were numerically prolonged in the atezolizumab/T-DM1 arm *vs.* placebo/T-DM1 arm: median PFS was 8.5 months *vs.* 4.1 months (HR 0.60, 95% CI 0.32–1.11), respectively, and 1-year OS was 94.3% *vs.* 87.9% (HR 0.55, 95% CI 0.22–1.38), respectively. Patients with PD-L1 positive tumor showed significantly higher TILs levels and the hazard ratio showed a larger benefit from atezolizumab/T-DM1 *vs.* placebo/T-DM1 in patients with TILs > 5% (HR 0.55, 95% CI 0.26–1.12) as compared to patients with lower TILs (HR 1.43, 95% CI 0.51–4.01). However, based on the small sample size and limited follow up these results warrant further evaluation in larger trials [37].

A plethora of other trials focused on anti PD-1/PD-L1/CTLA-4 antibodies in combination with anti-HER2 agents are ongoing and their results are awaited [52].

In HR+ MBC, the role of immunotherapy seems to be less promising and to date has not been largely explored.

In the multicohort phase Ib KEYNOTE-028 trial, pembrolizumab was investigated in 25 heavily pretreated PD-L1 positive HR+/HER2- MBC; ORR was 12% (95% CI 2.5–31.2%) with 12 months as median duration of response (range: 7.4–15.9 months). Overall, therapy was well tolerated but with modest response, even if durable in a subgroup of patients [53].

A phase II study randomizing HR+/HER2- heavily pre-treated MBC patients (≥ 2 previous lines of hormonal therapy and 0–2 previous lines of chemotherapy) to receive either the combination of eribulin-mesylate plus pembrolizumab (arm A) or eribulin-mesylate alone (arm B), reported no significant differences in terms of PFS (primary endpoint) and ORR (secondary endpoint). In particular, median PFS and ORR were 4.1 months *vs.* 4.2 months (*P* = 0.38), and 25% *vs.* 34% (*P* = 0.49), for arm A *vs.* arm B, respectively. Translational analyses failed to report a predictive role for PD-L1 status (PD-L1 positive tumors: > 1% with IHC 22C3 clone assay) TILs (cutoff 10%), TMB (cutoff 6), neutrophilic-lymphocyte ratio (cutoff 4) or genomic alterations with respect to immunotherapy efficacy [54].

Besides chemotherapy and anti-HER2 agents, there is growing interest in the identification of ideal partners for immune checkpoint blockade, with the aim of enhancing MBC patients’ prognosis. Many clinical trials testing combinations of ICIs with a variety of backbones agents [poly-ADP ribose polymerase (PARP)-inhibitors, anti-IDO (indoleamine 2,3 dioxygenase), CDK4/6 inhibitors], are currently ongoing. Interestingly, encouraging results have been reported with the combination of immunotherapy plus CDK 4/6 inhibitors [55, 56].

Preclinical evidence suggests that CDK 4/6 blockade may boost anti-tumor immune response by enhancing tumor antigen presentation (mediated by the activation of tumor cell expression of endogenous retroviral elements) as well as suppressing regulatory T-cell proliferation [57, 58].

In the JPCE phase 1b study, where 28 HR+/HER2- MBC with 1–2 prior chemotherapy regimens were treated with the combination of pembrolizumab plus abemaciclib, initial ORR was 14.3% and the rate of stable disease at 16 weeks was 60%. No association was observed between PD-L1 expression and response to abemaciclib plus pembrolizumab [59].

Encouraging results have also been reported from early-phase trials evaluating the combination of immunotherapy with PARP-inhibitors. Indeed, PARP-inhibition may lead to the accumulation of damages in the DNA, thus resulting in genomic instability [60].

In addition, it has been reported that PARP-inhibitors may upregulate PD-L1 expression in a preclinical model, resulting in T-cell activity attenuation. Conversely, the combination of PARP-inhibitors with PD-L1 blockade proved to restore T-cell killing activity, thus providing a strong rational to test combinations of PARP-inhibitors and ICIs in MBC patients [61].

In particular, MEDIOLA phase II basket trial included 34 HER2- (17 were TNBC) MBC patients harboring germline deleterious mutations of *BRCA* 1/2 genes. Patients were treated with the PARP-inhibitor olaparib for a 4-week run-in phase, which was then associated with the anti-PD-L1 agent durvalumab. Twelve-week (primary endpoint) and 28-week DCR were 80% (> target DCR of 75%) and 50%, respectively, while ORR (secondary endpoint) was 63.3% in the overall cohort. Higher ORR (70%) was observed in MBC patients treated with ≤ 1 prior line of chemotherapy than more heavily treated patients (ORR 50%). Interestingly, translational analyses revealed *BRCA2* reversion, TP53BP1 mutation, lack of *BRCA2* loss of heterozygosity, PD-L1 and PD-L2 amplification as possible biomarkers predicting treatment resistance [62].

In the single-arm TOPACIO phase II trial, 55 TN MBC (patients may have received ≤ 2 lines of therapy) received the combination of the PARP-inhibitor Niraparib plus pembrolizumab. ORR (primary endpoint) was 21% (90% CI 12–33%), DCR (secondary endpoint) was 49% (90% CI 36–62%), median PFS (secondary endpoint) was 8.3 months. Numerically higher response rates were observed in patients with tumor *BRCA* mutations (ORR 47%, 90% CI 24–70%) [63].

In both these trials the combination of PARP-inhibitors and immune checkpoint blockade was associated with a manageable safety profile.

Emerging evidence also suggests a possible role for immunotherapy as maintenance treatment, especially in TN MBC. Interestingly, the SAFIR-02 IMMUNOTRIAL trial evaluated durvalumab as maintenance therapy after 1st or 2nd line chemotherapy in 199 HER2- MBC not harboring any targetable molecular alteration [64]. Patients were randomly assigned 2:1 to either durvalumab or maintenance chemotherapy without switch. In the overall population, median PFS was 2.7 months with durvalumab *vs.* 4.6 months with chemotherapy HR 1.40 (95% CI 1.00–1.96, *P* = 0.047). In HR+/HER2- MBC patients, a detrimental effect with Durvalumab was observed as compared to chemotherapy (HR 2.08, 95% CI 1.28–3.40), whereas in TNBC patients the HR favored Durvalumab (0.87, 95% CI 0.54–1.42). In terms of OS, among TNBC patients, a significant improvement was observed with Durvalumab with respect to chemotherapy (median OS 21 months *vs.* 14 months, HR 0.54, 95% CI 0.30–0.97, *P* = 0.0377). Similarly, a trend towards a beneficial effect of Durvalumab has been observed in the PD-L1 positive cohort (> 1% of immune cells over tumor area by SP142 assay; median OS 26 months *vs.* 12 months, HR 0.42, 95% CI 0.17–1.05, *P* = 0.0552).

### Biomarkers

In addition to PD-L1 expression, other biomarkers are being studied for their ability to predict the benefit of immunotherapy in this setting.

TILs levels have consistently shown to be positively correlated with the level of PD-L1 expression [65–67].

In the KEYNOTE-086 trial of single-agent pembrolizumab, higher TILs were significantly associated with overall response rates in multivariable analysis [65].

Moreover, association between higher TILs and the efficacy of combined immunotherapy + anti-HER2 agents has been described in both the PANACEA and KATE2 trials [35, 36].

Tumor mutational burden also resulted associated with pembrolizumab efficacy vs chemotherapy in a biomarker analysis of the KEYNOTE-119 trial [68].

Copy number alterations (CNA) of PD-L1 (CD274) gene were evaluated in tumor tissues collected from 126 patients in the SAFIR-02 IMMUNOTRIAL. According to PD-L1 CNA, Durvalumab showed an OS improvement in the PD-L1 CNA gain/amplification subgroup (HR 0.17, 95% CI 0.05–0.55) with a median OS of 9 months in chemotherapy arm and not reached in durvalumab arm. This difference was particularly evident in TNBC patients (HR 0.18, 95% CI 0.05–0.71) [69].

Preliminary real-world data also suggest a role of body mass index (BMI) in affecting response to ICIs. Indeed, more favorable ORR and survival rates have been reported in obese/overweight patients with solid cancers receiving immunotherapy (proportion of BC patients, if any, has not been reported), thus suggesting immunotherapy as potentially capable of reverting the pro-tumorigenic immune dysfunction typically associated with overweight. However, definitive conclusions on the role of BMI in BC patients receiving immunotherapy is currently unknown [70, 71].

## Conclusion

Immunotherapy is emerging as a new frontier in BC treatment, leading to a new standard of care for the first line treatment of TN MBC with PD-L1 positive status. Despite this breakthrough, only a minority of BC patients seems to benefit from this novel treatment strategy. In this context, there is increasing interest in identifying the ideal partner for ICIs. The most promising results have been achieved by associating immunotherapy with chemotherapy, possibly due, at least in part, to the mechanism of chemotherapy-induced immunogenic cell death [72, 73]. However, results from early-phase clinical trials testing the combination with various biological agents, like anti-HER2 agents, PARP-inhibitors and CDK 4/6 inhibitors are encouraging, thus potentially allowing to broaden the population of BC patients who may benefit from immunotherapy. In addition, several trials currently testing combinations with other targeted agents are ongoing and results are awaited [74].

Other unresolved issues need to be addressed. First, a comprehensive predictive biomarker for immunotherapy efficacy capable of reliably identifying BC patients who may benefit from immunotherapy both in advanced and early setting is still lacking. In this context, although PD-L1 expression is associated with immunotherapy benefit in different trials in the metastatic setting, several other clinical studies failed to report a similar predictive role and there is a major discrepancy with results obtained by neoadjuvant trials. Moreover, the dynamic changes and heterogeneity of PD-L1 expression in the same sample tumor and in the different metastatic sites, together with the absence of reliable detection methods, limit its use in clinical practice. Other potential biomarkers of response/resistance to immunotherapy such as TILs, TMB, characterization of the immune infiltrate or gut microbiome have been investigated and may will provide additional information [35, 43, 44, 66, 75–77]. Unfortunately, these biomarkers require specific analyses in tumor specimens, which are not always easily available. Therefore, cheap and easy-to-measure biochemical/clinical parameters could significantly help in patients’ selection, like lactate dehydrogenase (LDH) [78] and BMI [70, 71].

Lastly, although available evidence is consistent in suggesting a larger beneficial effect of immunotherapy when administered earlier in the natural history of BC, the ideal timing for immunotherapy is still object of debate [42, 44–46]. The long journey of immunotherapy in BC has just started and its full potential yet to be discovered.

## Abbreviations

AC: doxorubicin-cyclophosphamide
BC: breast cancer
CDDP: cisplatin
CNA: copy number alterations
CPS: combined positive score
CTX: cyclophosphamide
DCR: disease control rate
EBC: early breast cancer
EC: epirubicin-cyclophosphamide
EFS: event-free survival
EMA: European Medicines Agency
HER2-: HER2-negative
HER2+: HER2-positive
HR-: hormone receptor-negative
HR+: hormone receptor-positive
ICI: immune checkpoint inhibitor
iTILs: intra-tumoral tumor-infiltrating lymphocytes
ITT: intention-to treat population
mAb: monoclonal antibody
MBC: metastatic breast cancer
NabP: nab-paclitaxel
OR: odds ratio
ORR: objective response rate
OS: overall-survival
PARP: poly-ADP ribose polymerase
pCR: pathologic complete response
PD-L1: anti-programmed death-ligand 1
PFS: progression free survival
sTILs: stromal tumor-infiltrating lymphocytes
TILs: tumor infiltrating lymphocytes
TMB: tumor mutational burden
TN: triple-negative
TNBC: triple-negative breast cancer

## Declarations

### Copyright

© The Author(s) 2020.
